# Hepatic inflammatory myofibroblastic tumor: A case report and literature review

**DOI:** 10.1097/MD.0000000000043739

**Published:** 2025-08-01

**Authors:** Jinyu Liu, Gang Wang, Ting Ge, Xingdong Xu

**Affiliations:** a Department of Hepatobiliary Surgery, The First College of Clinical Medical Science, China Three Gorges University and Yichang Central People’s Hospital, Yichang, Hubei Province, China.

**Keywords:** inflammatory myofibroblastic tumor, inflammatory pseudotumor, liver

## Abstract

**Rationale:**

Hepatic inflammatory myofibroblastic tumor (HIMT) is a rare intermediate neoplasm, characterized by localized proliferation and low metastatic potential. Despite its benign nature, HIMT exhibits malignant features including recurrence and multifocal growth. The etiology of HIMT is multifactorial and involves infections, trauma, surgery, and radiotherapy. A history of biliary surgery or infection is strongly associated with the development of HIMT.

**Patient concerns:**

A 48-year-old female presented with 2-week fatigue (eastern cooperative oncology group 1) and weight loss of 4 kg.

**Diagnoses:**

Computed tomography and magnetic resonance imaging resonance imaging revealed a tumorlike lesion in the right posterior lobe of the liver.

**Interventions:**

The patient underwent an open right hemihepatectomy without adjuvant therapy.

**Outcomes:**

Immunohistochemistry confirmed HIMT: smooth muscle actin+, anaplastic lymphoma kinase-, CD21-, and S-100-. No perioperative complications or recurrence was observed during the 36-month follow-up period.

**Lessons:**

HIMT is a rare neoplasm that is often misdiagnosed owing to its nonspecific presentation. Surgical resection remains the primary treatment; however, standardized therapeutic protocols are required.

## 1. Introduction

Inflammatory myofibroblastic tumor (IMT), formerly known as “inflammatory pseudotumor,” is a rare clonal mesenchymal neoplasm of intermediate biological potential, defined by proliferating myofibroblastic spindle cells admixed with lymphoplasmacytic infiltrates.^[1]^ While predominantly occurring in the lungs and abdominopelvic regions, hepatic IMT (HIMT) is exceptionally rare, comprising < 1% of liver masses.^[2]^ Its pathogenesis remains incompletely understood but may involve prior surgery, infection, or trauma. Molecularly, approximately 60% of HIMTs feature anaplastic lymphoma kinase (ALK) rearrangements, particularly in younger patients.^[3]^ ALK-negative tumors frequently harbor fusions in C-ros oncogene 1 receptor tyrosine kinase (ROS1), neurotrophic receptor tyrosine kinase 3 (NTRK3), or rearranged during transfection and demonstrate more aggressive behavior with poorer therapeutic responses.^[4–6]^ HIMT is often misdiagnosed as hepatocellular carcinoma, cholangiocarcinoma, or abscess due to overlapping clinical and radiological features. Complete surgical resection is the curative mainstay; molecular profiling guides targeted inhibitor therapy for unresectable or recurrent disease.^[7,8]^ We present an ALK-negative HIMT in a middle-aged female, illustrating diagnostic challenges, the evolving molecular landscape, and therapeutic implications.

## 2. Case report

A 48-year-old female presented with 2-week fatigue (eastern cooperative oncology group 1) and 4 kg weight loss. The patient presented in good condition, with no significant past medical history. Abdominal examination revealed no tenderness or rebound tenderness, and no abnormal masses were palpated. Laboratory tests revealed leukocytosis (13.85 × 10^9^/L) with neutrophilia (84.9%), elevated CRP (129.3 mg/L), and anemia (hemoglobin 75 g/L). Liver function tests showed elevated GGT (344 U/L), AST (64 U/L), and ALT (81 U/L), with hypoalbuminemia (26.1 g/L). Tumor markers (AFP, CA19-9, and CEA) and serological tests for hepatitis and Echinococcus were negative.

**Figure 1.**
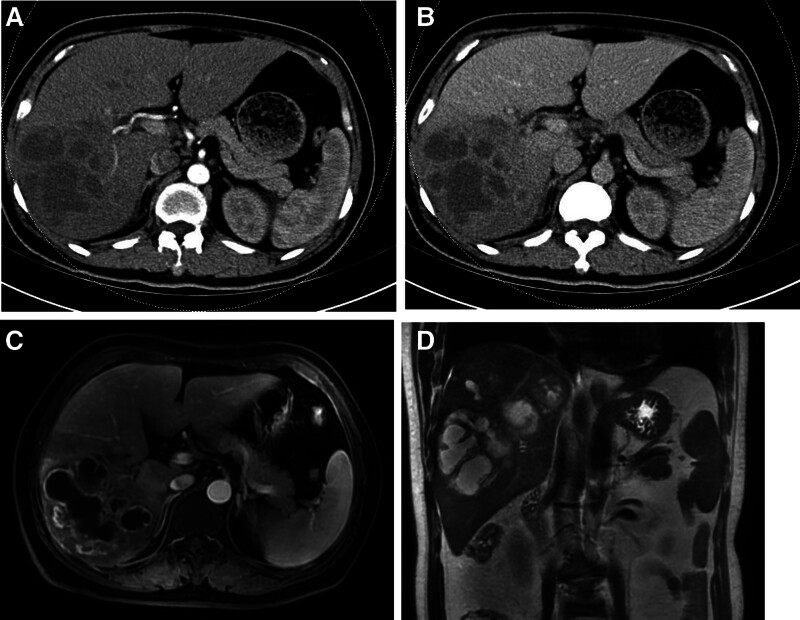
Contrast-enhanced CT (A, B) and MRI (C, D) demonstrating a solid-cystic hepatic mass. CT = computed tomography, MRI = magnetic resonance imaging.

**Figure 2.**
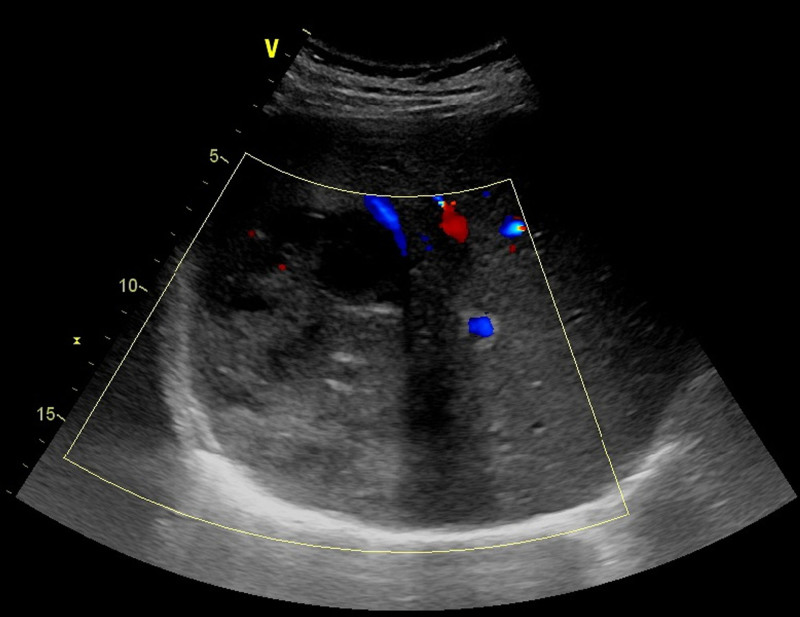
Ultrasonography images of the hepatic inflammatory myofibroblastic tumor.

Contrast-enhanced computed tomography (CT) revealed a 135 × 120 × 100-mm solid-cystic mass in the right posterior liver lobe with peripheral enhancement (Fig. 1A, B). Abdominal magnetic resonance imaging (MRI) demonstrates a 105 × 72 × 128-mm mass in the right posterior hepatic lobe exhibiting mild T1 hypointensity, marked T2 hyperintensity, progressive delayed enhancement of the pseudocapsule and internal septa, along with central necrosis. Imaging features are suggestive of atypical hepatocellular carcinoma; pyogenic abscess remains a differential consideration (Fig. 1C, D). Color Doppler ultrasound demonstrates a 9.5 × 8.8-cm mixed-echogenicity mass in the right posterior hepatic lobe, predominantly hypoechoic with peripheral vascularity (Fig. 2). The patient’s liver function was classified as Child-Pugh class A with a future liver remnant volume > 40%. The patient underwent open right hemihepatectomy, with postoperative histopathology confirming hepatic inflammatory myofibroblastic tumor (HIMT).

**Figure 3.**
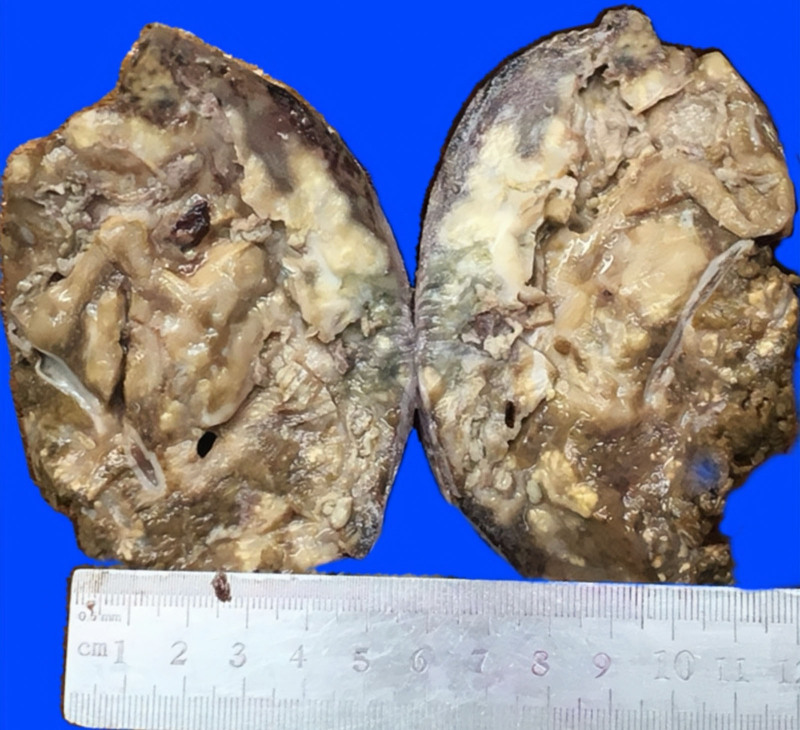
Gross appearance of the resected tumor.

**Figure 4.**
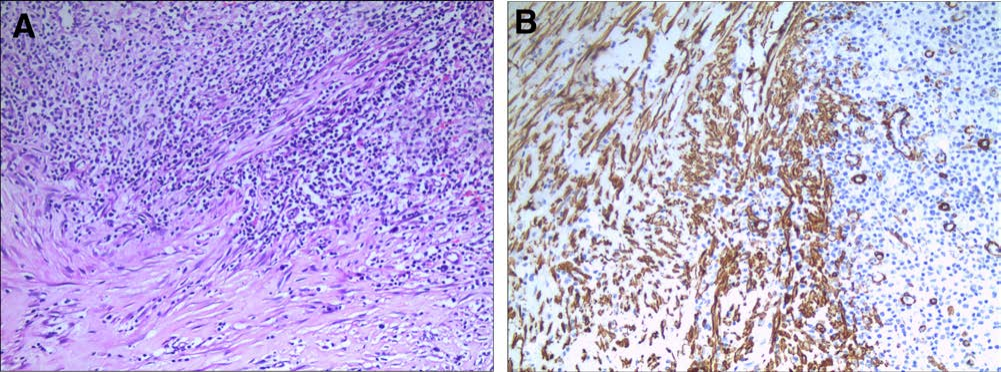
Histopathology (A: H&E staining, 400×; B: SMA positivity, 400×). SMA = smooth muscle actin.

Gross examination revealed an 8 × 6 × 6-cm mass with a grayish-brown cut surface and indistinct margins (Fig. 3). Histopathology demonstrated spindle cell proliferation with plasma cells, lymphocytes, and neutrophils on a myxoid and collagenous background (Fig. 4A). Immunohistochemistry showed smooth muscle actin positivity and focal desmin expression, whereas ALK, CD21, and S-100 were negative, confirming hepatic IMT (Fig. 4B). The patient recovered uneventfully with no recurrence at 36 months.

## 3. Discussion

IMT can occur in almost all solid organs, with the lungs being the most common site (25%), followed by the mesentery and omentum. Occurrences in the liver and bile ducts are relatively rare.^[9]^ HIMT is an extremely rare condition that typically affects young individuals. The pathogenesis of HIMT is unclear and may be associated with factors, such as infection, hormones, and trauma.^[10]^ History of biliary surgery or infection was particularly significant. Hepatolithiasis associated with anatomical anomalies can lead to recurrent biliary tract infections and may be a contributing factor.^[11]^ Emerging evidence suggests that inflammatory triggers may induce myofibroblastic proliferation, leading to tumor formation.

HIMT presents with nonspecific symptoms including fatigue, weight loss, and abdominal pain, often leading to misdiagnosis. Laboratory tests for HIMT were nonspecific, with normal liver function and negative HBsAg and AFP levels. Elevated AFP levels in some cases may be related to IMT stimulating hepatocyte proliferation or concurrent active liver disease. The patient in this case report was a middle-aged woman who was found to have fatigue and weight loss.

Radiologically, HIMT is predominantly located at the liver margin or beneath the capsule, forming a mass due to the replacement of normal liver tissue with fibroblastic proliferation and inflammatory infiltration. On CT imaging, HIMT typically appears as a hypodense lesion, reflecting replacement of the hepatic parenchyma by fibroblastic cells and inflammatory infiltrates. MRI findings often reveal lesions with irregular borders and heterogeneous signal intensity.^[12]^ On T1-weighted imaging, HIMT exhibits slightly hypointense signals, whereas T2-weighted imaging demonstrates hyperintense signals. The signal intensity on T2-weighted imaging correlates with the degree of interstitial edema and mucinous collagen degeneration; higher signals indicate more edema and degeneration, whereas lower signals suggest a predominance of spindle cells.^[13]^ In the arterial phase of contrast-enhanced CT and MRI, HIMT typically shows minimal or no enhancement, likely because of its predominant portal venous supply and lack of hepatic arterial perfusion. During the portal venous and delayed phases, the lesion exhibited gradual, persistent, and delayed enhancement patterns.^[14]^ Additionally, some HIMT cases display the “vascular floating sign” (also termed the “hanging fruit sign”), characterized by portal vein branches traversing the lesion during the portal venous phase.^[15]^ This sign, which is not fully understood, is considered relatively specific for HIMT and may result from contrast retention within the neovascular bundles and inflammatory cell clusters. In the present case, the patient’s CT scan revealed a hypodense lesion with no significant arterial phase enhancement, consistent with the absence of a hepatic arterial supply.

The diagnosis of HIMT requires comprehensive analysis of histomorphological, immunophenotypic, and molecular genetic features. Grossly, HIMT typically presents as an ill-defined mass with a grayish-white or yellowish cut surface and firm texture, often blended with the surrounding hepatic parenchyma. These nonspecific gross features provide critical clues for histopathological evaluations. Histologically, HIMT is classified into 3 subtypes: myxoid vascular, hypocellular fibrous, and spindle cell proliferative.^[3]^ These components often coexist and are accompanied by coagulative necrosis. Hemorrhage, necrosis, and calcification are uncommon, although dystrophic calcification or ossification is occasionally observed.^[16]^ Immunohistochemical analysis revealed myofibroblastic differentiation in HIMT.^[17]^ Approximately 80% to 90% of cases express smooth muscle actin, a key diagnostic marker.^[18]^ Muscle-specific actin, desmin, and calponin are positive in 60% of cases.^[19]^ Molecular genetic alterations not only aid in diagnosis, but also provide prognostic insights, particularly in ALK-negative cases. Approximately 60% of HIMT cases express ALK, with ALK positivity being more common in younger patients and associated with gene rearrangements.^[20]^ The subcellular localization of ALK is determined by its fusion partner, and the resulting fusion gene activates downstream signaling pathways, promoting cell proliferation and tumorigenesis.^[21]^ Thus, ALK is considered a significant diagnostic marker for HIMT.^[22]^ Recent studies suggest that ALK-negative HIMT may harbor alternative fusion genes, such as ROS1 and NTRK3, which are associated with aggressive behavior and are potential therapeutic targets.^[23]^ These findings offer new directions for the diagnosis and treatment of ALK-negative HIMT. Here, we report a case of ALK-negative HIMT.

Currently, there are no unified treatment guidelines for HIMT, and treatment options include surgery and conservative and observational therapies. Surgical resection remains the primary treatment for HIMT, with a recurrence rate of 19% to 37%.^[5,24]^ Complete tumor removal is critical for minimizing recurrence. ALK fusion genes have been proven to be therapeutic targets, and ALK inhibitors have been widely reported for the treatment of ALK-positive cases, while targeted therapies against ROS1 or NTRK3 fusions offer promise for ALK-negative tumors.^[25,26]^ Conservative treatments, including corticosteroids and nonsteroidal anti-inflammatory drugs, are viable options for patients with contraindications for surgery.^[27]^ In most cases, owing to the lack of a clear preoperative diagnosis or disease progression, almost all patients with IMT have undergone radical surgery.^[28]^ Genetic testing will help in selecting the appropriate treatment plan for patients with HIMT who cannot undergo surgery. The patient in this case underwent surgical treatment, with no serious complications in the perioperative period and has not recurred to date.

The prognosis of HIMT is generally favorable, with low metastatic potential.^[29]^ However, ALK-negative cases may exhibit a more aggressive behavior, necessitating close follow-up. Molecular profiling is becoming increasingly important for guiding treatment decisions, particularly in cases where surgical resection is not feasible.

## 4. Conclusion

HIMT is a rare neoplasm requiring prompt diagnosis and tailored treatment. Although surgical resection remains the cornerstone, molecular-targeted therapies offer new hope for ALK-negative patients.

## Author contributions

**Data curation:** Jinyu Liu, Gang Wang, Ting Ge.

**Writing – review & editing:** Xingdong Xu.
